# Practice patterns in chronic graft-versus-host disease patient management and patient reported outcome measures across the EBMT allogeneic transplantation network

**DOI:** 10.1038/s41409-022-01733-3

**Published:** 2022-06-11

**Authors:** Vladimir Perovic, Ivan Sabol, Magdalena Grce, Marit Inngjerdingen, Drazen Pulanic, Zinaida Peric, Christophe Peczynski, Emmanuelle Polge, Christian Koenecke, Anne Dickinson, Hildegard Greinix, Grzegorz Basak, Olaf Penack, Angela Scherwath, Anna Barata, Attilio Olivieri, Anita Lawitschka, Patrycja Mensah-Glanowska, Hajnalka Andrikovics, Helene Schoemans, Daniel Wolff

**Affiliations:** 1 Institute for Microbiology and Immunology, Faculty of Medicine, University of Belgrade, Belgrade, Serbia; 2 Division of Molecular Medicine, Ruđer Bošković Institute, Zagreb, Croatia; 3 Department of Pharmacology, Institute of Clinical Medicine, University of Oslo and Oslo University Hospital, Oslo, Norway; 4 Division of Hematology, Department of Internal Medicine, University Hospital Center Zagreb, Zagreb, Croatia; 5 School of Medicine, University of Zagreb, Zagreb, Croatia; 6 Clinical Sorbonne University Hematology and Cellular Therapy, Saint Antoine Hospital, INSERM UMR 938, Sorbonne University, Paris, France; 7 Department of Hematology, Hemostasis, Oncology and Stem Cell Transplantation, Hannover Medical School, Hannover, Germany; 8 Translational and Clinical Research Institute, Faculty of Medical Sciences, Newcastle University, Newcastle upon Tyne, United Kingdom; 9 Division of Hematology, Department of Internal Medicine, Medical University of Graz, Graz, Austria; 10 Department of Hematology, Oncology and Internal Medicine, Medical University of Warsaw, Warsaw, Poland; 11 Charité – Universitätsmedizin Berlin, corporate member of Freie Universität Berlin and Humboldt-Universität zu Berlin, Department of Department of Hematology, Oncology and Tumorimmunology, Berlin, Germany; 12 Department of Stem Cell Transplantation, University Medical Center Hamburg-Eppendorf, Hamburg, Germany; 13 Health Outcomes and Behavior Program, Moffitt Cancer Center, Tampa, FL USA; 14 Hematology Department, Hospital de la Santa Creu i Sant Pau, Barcelona, Spain; 15 Joseph Carreras Leukemia Research Institute, Barcelona, Spain; 16 Hematology Department and Stem Cell Transplant Unit, Università Politecnica delle Marche Università Politecnica delle Marche, Ancona, Italy; 17 Stem Cell Transplantation (SCT)-Outpatient and Aftercare Clinic, St. Anna Children’s Hospital, Medical University Vienna, Vienna, Austria; 18 Jagiellonian University Medical College / University Hospital, Krakow, Poland; 19 Laboratory of Molecular Genetics, Central Hospital of Southern Pest, National Institute of Hematology and Infectious Diseases, Budapest, Hungary; 20 Department of Public Health, University Hospitals of Leuven and KU Leuven, Leuven, Belgium; 21 Department of Internal Medicine III, Faculty of Medicine, University Hospital Regensburg, Regensburg, Germany

**Keywords:** Haematological diseases, Public health

## To the Editor:

## Background

Chronic graft-versus-host disease (cGvHD) is one of the most common life-threatening complications following allogeneic haematopoietic stem cell transplantation (alloHSCT). Understanding outcome after alloHSCT requires a full evaluation of the patient’s health status, including cGvHD and patient reported outcomes (PROs). In an effort to better understand practice patterns across European countries, a survey was initiated by the Integrated European Network on cGvHD (an EU-funded COST Action CA17138 EUROGRAFT, www.gvhd.eu) and the Transplant Complications Working Party of the European Society for Blood and Marrow Transplantation (EBMT). This report shares results of the survey, offering a snapshot view of current practice patterns in the context of long-term care of cGvHD patients.

## Methods

Our self-designed 38-item online survey (Supplementary Material) was intended to collect data regarding transplant center characteristics, data registration practices, the use of NIH criteria in clinical routine, biopsies/biomarkers for clinical assessment, cGvHD cell-based therapies, and PROs. The survey used computer adapted testing methods and took ~10 min to complete. All centers participating in the COST Action EUROGRAFT and all EBMT centers performing alloHSCT were invited by email for participation in the survey. Data were collected between July 2019 and July 2020. Appropriate descriptive statistics were used. In case of multiple entries for a single center (*n* = 4), only the entry from the most senior staff member was included for the analysis. Missing data was reported as such.

## Findings

### Center characteristics

Survey results are summarized in Table 1. A total of 72 centers out of 424 invited centers from 24 countries responded to the survey, representing ~17% of all alloHSCT centers and 19.6% of all transplanted patients within the EBMT network [1]. The majority of participating alloHSCT centers were from Europe with exception of three centers based in Asia and one in Latin America. Survey responses were mainly submitted by physicians and data managers. Of note, the size of the transplant programs differed between responding (mean ± SD, *n* = 47 ± 40 transplants/year) vs. non-responding (mean ± SD, *n* = 39 ± 31 transplants/year) centers (Supplementary Material).

**Table 1:** Summary results of the survey.

Center responses	*N* (%)
Total centers responding	72 (17.0)
Data registration	
Use own registry outside of EBMT	65 (90.3)
Use of NIH criteria	68 (94.4)
Routinely use NIH criteria for diagnosis and severity grading outside of clinical trials^a^	
NIH2005	13 (19.1)
NIH2014	6 (8.8)
Both	49 (72.1)
Using NIH response criteria outside clinical trials	37 (51.4)
Use of Biomarkers	
Confirming skin, ocular and oral mucosa cGvHD by histopathology:	
Rarely (<10%)	26 (36.1)
Sometimes (10–39%)	16 (22.2)
Often (40–74%)	17 (23.6)
Routinely (>75%)	7 (9.7)
Use of specific biomarkers	5 (6.9)
Collecting and storing patient samples^a^	22 (30.6)
At calendar-driven time points	15
At the onset of cGvHD	10
During the treatment of cGvHD	7
Before transplantation	3
Use of PROs	22 (30.6)
Setting of use of PROs^a^:	
As integral part of the clinical evaluation	20
As part of the outcome analysis	13
As monitoring of response to treatment	13
For referral to specialists	9
Most common reasons for not using PROs^a^:	
Resource constraints	36
Time constraints	33
Not available in the required language	14
Not familiar with interpretation of PRO data	7
Additional burden for the patients	5
Types of questionnaires used^a^	
Standardized	18
Questionnaires developed for specific clinic or research purposes	11
Types of standardized questionnaires used^a^:	
FACT-BMT	6
Lee chronic GvHD symptom scale	6
NIH Form B	5
SF-36	4
EORTC QLQ-C30	3
EQ5D	3
Other	10
Use of cell-based cGvHD therapies^a^	39 (54.2)
MSCs	27
ECP closed system	24
ECP open system	19
Tregs	5

*PRO* patient reported outcomes, *MSC* mesenchymal stromal cells, *ECP* extracorporeal photopheresis, *Tregs* regulatory T cells.

^a^Denotes that several answers were possible.

### Chronic GvHD patient diagnosis and management

Over 80% of respondents reported that post-transplant care was provided by multidisciplinary teams comprised of clinicians including subspecialists such as pulmonologists, gynecologists, ophthalmologists, and dermatologists. The majority of responding centers (*n* = 65; 90.3%) used their own database for collecting and storing patient information. Almost all participating centers (*n* = 68; 94.4%) reported using NIH consensus criteria on cGvHD diagnosis and severity grading, while 51% of responders used NIH response criteria outside of the context of clinical trials. The top three reasons for not using these criteria were their complexity, the lack of suitability for use in children as well as time constraints.

Only a small fraction of centers, (*n* = 5; 6.9%) used specific biomarkers (e.g., Reg3-alpha, ST2, CXCL9 etc.) in the context of cGvHD. Collection and storage of patient samples for future assessment of biomarkers was not a common practice either, with less than one third of centers (22/72) collecting patient samples. Approximately 54.2% (*n* = 39) of responding centers reported using cell-based therapies for treatment of cGvHD. Most frequently used therapies were mesenchymal stromal cells (MSCs) (*n* = 27), extracorporeal photopheresis (ECP) closed system (*n* = 24) and ECP open system (*n* = 19). In contrast, cell-based therapies were rarely used in prophylaxis (*n* = 4; 10.2%).

### Collection and use of PROs

The collection of PROs in routine practice was limited (*n* = 22; 30.6%). When used, PRO questionnaires were essentially used as an integral part of clinical evaluation and mainly administered to patients using paper and pencil (*n* = 19; 86.4%). Standardized questionnaires or questionnaires developed for specific clinic or research purposes were both used for PRO data acquisition. The most common PRO measures used were the Lee cGvHD symptom scale [2] (*n* = 6), the FACT-BMT (*n* = 6) and the NIH Form B (*n* = 5). Notably, centers reporting use of PROs frequently collaborated with patient associations/support/advocacy groups (*n* = 12; 63.2%). Lack of time and lack of resources were the most common barriers for data collection.

## Discussion

With the improvement of transplantation outcome [3] and the growing number of alloHSCT survivors [4], chronic diseases such as cGvHD are becoming a growing concern of healthcare systems. Our survey illustrates the current trends in cGvHD management and use of PROs in alloHSCT centers across the EBMT and COST Action EUROGRAFT network, highlighting a high uptake of NIH criteria in routine practice, going beyond the initial evaluation of Duarte et al. [5]. Despite recent advances in the field [6], the use of biomarkers and cellular therapies for cGvHD remain modest. Finally, while already 17% of replying centers indicated the use of PRO´s in clinical routine, their integration in clinical care should be promoted based on current recommendations indicating their validity in including the patient’s perspective in transplantation outcome evaluation [7–9].

## Conclusions

The interpretation of this survey needs to take into account the limited response rate and the risk of a potential responder bias since information was likely provided mainly by centers with a particular interest in cGvHD and long-term care. In line with this, the observed difference in the size of the transplant programs between responding and non-responding centers may explain, at least in part, reported results of limited use of PROs and biomarkers. Larger and/or more experienced alloHSCT centers may have better infrastructure and resources than centers with smaller transplant programs for real-world implementation of NIH response criteria. Our results might therefore not be fully representative of common practice across Europe. Nevertheless, this survey highlights the need for the harmonization of current cGvHD management practices. This underpins one of the aims of COST Action EuroGraft to expand collaboration between European transplant centers and provide training for transplant programmes to foster a harmonized approach for diagnosis and treatment of cGvHD. This COST Action is offering a new platform to develop common initiatives in cGvHD management to optimize long-term outcome of alloHSCT patients.

## Supplementary information

Table 1 supplement

Survey

## Data Availability

Original data can be shared at request.

## References

[1] Passweg JR, Baldomero H, Basak GW, Chabannon C, Corbacioglu S, Duarte R (2019). The EBMT activity survey report 2017: a focus on allogeneic HCT for nonmalignant indications and on the use of non-HCT cell therapies. Bone Marrow Transpl.

[2] Lee SJ, Wolff D, Kitko C, Koreth J, Inamoto Y, Jagasia M (2015). Measuring therapeutic response in chronic graft-versus-host disease. National Institutes of Health consensus development project on criteria for clinical trials in chronic graft-versus-host disease: IV. The 2014 Response Criteria Working Group report. Biol Blood Marrow Transpl.

[3] Penack O, Peczynski C, Mohty M, Yakoub-Agha I, Styczynski J, Montoto S (2020). How much has allogeneic stem cell transplant-related mortality improved since the 1980s? A retrospective analysis from the EBMT. Blood Adv.

[4] Majhail NS, Tao L, Bredeson C, Davies S, Dehn J, Gajewski JL (2013). Prevalence of hematopoietic cell transplant survivors in the United States. Biol Blood Marrow Transpl.

[5] Duarte RF, Greinix H, Rabin B, Mitchell SA, Basak G, Wolff D (2014). Uptake and use of recommendations for the diagnosis, severity scoring and management of chronic GVHD: an international survey of the EBMT-NCI Chronic GVHD Task Force. Bone Marrow Transpl.

[6] Crossland RE, Perutelli F, Bogunia-Kubik K, Mooney N, Milutin Gasperov N, Pucic-Bakovic M (2020). Potential novel biomarkers in chronic graft-versus-host disease. Front Immunol.

[7] Burns LJ, Abbetti B, Arnold SD, Bender J, Doughtie S, El-Jawahiri A (2018). Engaging patients in setting a patient-centered outcomes research agenda in hematopoietic cell transplantation. Biol Blood Marrow Transpl.

[8] Bevans M, El-Jawahri A, Tierney DK, Wiener L, Wood WA, Hoodin F (2017). National institutes of health hematopoietic cell transplantation late effects initiative: the patient-centered outcomes working group report. Biol Blood Marrow Transpl.

[9] Schoemans HM, Finn L, Foster J, Roche-Green A, Bevans M, Kullberg S (2019). A conceptual framework and key research questions in educational needs of blood and marrow transplantation patients, caregivers, and families. Biol Blood Marrow Transpl.

