# Supplementary material for: Practice patterns in chronic graft-versus-host disease patient management and patient reported outcome measures across the EBMT allogeneic transplantation network
Source: Bone Marrow Transplant. 2022 Jun 11;57(9):1458–60. doi: 10.1038/s41409-022-01733-3 (PMC9439950; doi:10.1038/s41409-022-01733-3)
Supplement: Supplementary file 1 — Table 1 supplement [file 41409_2022_1733_MOESM1_ESM.docx]

|  | Responding centers | | Non responding centers | | |
| --- | --- | --- | --- | --- | --- |
| *# of transplants/year* | *#* | % | *#* | | *%* |
| 1-10 | 4 | 5.50% | 48 | 14.16% | |
| 11-20 | 12 | 16.70% | 63 | 18.60% | |
| 21-40 | 20 | 27.80% | 100 | 29.50% | |
| 41-80 | 26 | 36.10% | 95 | 28.00% | |
| 81-100 | 7 | 9.70% | 16 | 4.70% | |
| > 100 | 3 | 4.20% | 17 | 5.00% | |
| Mean ±SD |  | 46.75±39.90 |  | 38.9 ±31.04 | |
| Median (IQR range) |  | 40.5 (22.5-57.75) |  | 29.5 (16.5-53.5) | |
| Total | 72 |  | 339* |  | |

Table 2. Comparison between responding and non responding alloHSCT centers

*Data were obtained from EBMT activity survey report 2017 representing the number of first allogeneic HCT per year [1]. *Data for 13 alloHSCT centers were not available.*
