# Supplementary material for: Practice patterns in chronic graft-versus-host disease patient management and patient reported outcome measures across the EBMT allogeneic transplantation network
Source: Bone Marrow Transplant. 2022 Jun 11;57(9):1458–60. doi: 10.1038/s41409-022-01733-3 (PMC9439950; doi:10.1038/s41409-022-01733-3)
Supplement: Supplementary file 2 — Survey [file 41409_2022_1733_MOESM2_ESM.docx]

CA17138 - EUROGRAFT survey

CA17138 - EUROGRAFT survey about data collection after hematopoietic stem cell transplantation, with a specific focus on chronic GVHD

The Integrated European Network on Chronic Graft Versus Host Disease (cGVHD) is a new EU-funded COST Action initiated to overcome the lack of coordination at the European research level regarding chronic GVHD diagnosis and therapy. This survey is sponsored by this initiative and performed in collaboration with the Transplant Complications working party of the EBMT (European Society for Blood and Marrow transplantation).

This survey aims at exploring current practice patterns in Europe with regard to:
- GVHD data collection after hematopoietic stem cell transplantation (HCT)
- collection and use of biomarkers
- data collection and use of Patient Reported Outcomes (PRO), such as Quality of Life (QoL)
- use of NIH criteria in clinical routine
- application of cellular therapy in prophylaxis and treatment of GVHD

This information will help to improve the understanding and management of chronic GVHD across European countries.

Filling in this survey takes approximately 10 minutes. Responses will be kept confidential.
We kindly ask you to provide answers reflecting your center’s policy.


We really appreciate your collaboration!

These answers describe the institution and the person responding to the survey.

A) BMT Centre identification name *

B) EBMT CIC code (if applicable)

C) Country *

D) Respondent’s full name (optional)

E) E-mail (optional):

F) Respondent’s responsibility with HCT program: *

- Head of department
- Head of HCT program
- HCT Clinician
- Data manager
- Quality manager
- Clinician (not primary active in HCT)
- Nurse
- Other:

General part

G) What is the clinic arrangement for cGVHD patients in your center (please check all that apply) *

- Each provider cares for his/her own patients
- Dedicated cGvHD clinic / multidisciplinary team
- Long term follow-up clinic, including cGvHD clinic / multidisciplinary team
- Other:

H) What specialists are available in the care of cGVHD patients in your center (please check all that are available) *

- Dental care / oral medicine
- Dermatology
- Gynecology
- Infectious disease
- Neurology
- Nutrition
- Ophtalmology
- Pathology
- Physical therapy
- Psychiatry
- Psychology
- Pulmonary
- Urology
- None
- Other:

The questionaire consists of 9 specific questions describing the collection and use of cGVHD relevant data and material. Some of the questions have sub-questions that are not show if not applicable.

1. Does your centre collect clinical medical data of allogeneic hematopoietic stem cell transplantation (alloHCT) patients for your own or other registries outside of EBMT registry data? *

- Yes
- No

1.1. If YES, in which patients: *

- In all alloHCT patients
- In alloHCT patients with GVHD only
- In alloHCT patients with acute GVHD only
- In alloHCT patients with chronic GVHD only

2. Does your centre routinely use the NIH criteria for diagnosis and severity grading outside clinical trials? *

- Yes
- No

2.1 If YES, which criteria? *

- NIH 2005
- NIH 2014
- Both

2.2. If NO, please indicate the reason (check all that apply): *

- Lack of knowledge
- Lack of clinical relevance
- Time constraints
- Other:

3. In patients with suspected de novo chronic GvHD involving the skin, oral mucosa and eyes but lacking diagnostic criteria (i.e. maculopapular rash involving 40% of the body surface and mild sicca-symptoms of the oral mucosa and eyes at day 200, 8 weeks after stop of immunosuppression) how often do you confirm presence of GVHD by histopathology? *

- >75%
- 40-74%
- 10-39%
- <10%

4. Does your centre use the official NIH response criteria outside clinical trials? *

- Yes
- Sometimes
- No

4.1 If YES/SOMETIMES, which criteria? *

- NIH 2005
- NIH 2014
- Both

5. Does your centre analyse specific biomarkers (like Reg3-alpha, ST2, CXCL9, other) in the context of GVHD (in addition to routine diagnostic like platelets)? *

- Yes
- No

5.1. If YES, in which patients: *

- Only acute
- Only chronic
- Both

5.2. If YES, in which context: *

- In research projects
- In clinical routine
- Both

6. Does your centre store patient samples for potential assessment of biomarkers (serum, plasma, urine, MNC, DNA) along with clinical data? *

- Yes
- No

6.1. If YES, which time points (select all that apply)? *

- Around day 28
- Around day 90
- At the end of immune suppression
- At the onset of cGVHD
- During the treatment of cGVHD
- Other:

6.2. If YES, which samples (select all that apply)? *

- DNA
- Serum
- Plasma
- Urine
- Faeces
- MNC´s
- Other:

6.3. Do you have informed consent for sharing and additional analysis? *

Is your center allowed to share collected patient samples with other researchers for analysis of initially unforeseen biomarkers

- Yes
- No

7. Does your centre collect Patient Reported Outcomes (PROs) such as QoL data in alloHCT patients? *

- Yes
- No

7.1. If NO, why do you not collect PRO data? (please check all that apply) *

- Time constraints
- Resource constraints
- Additional burden for the patients
- Not relevant for treatment decision
- Not familiar with interpretation of PRO data
- Not available in the required language
- Other:

7.2. In which context do you collect PRO/QoL data? (please check all that apply) *

- Routine registration of your own centre
- Routine registration in a national register (please specify the name of the register you participate in under OTHER)
- Clinical trials or multicentre studies
- Other:

Referring to the use of routine PRO data in clinical practice (NOT in clinical trials you might be participating in) please answer the following questions:

7.3. Do you collect routine PRO data in: *

- Adults only
- Children only
- Both, adults & children

7.4. In which patient subgroups do you collect PRO data? *

- In all alloHCT patients
- In alloHCT patients with GVHD only
- In alloHCT patients with acute GVHD only
- In alloHCT patients with chronic GVHD only
- Other:

7.5. How do you collect routine PRO data in your HCT patients? (please check all that apply) *

- I use standardized questionnaires
- I use a tool/system developed in our centre
- Other:

7.6. Which standardized instruments do you use to collect routine PRO data in your BMT patients? (please check all that apply) *

- SF-36
- EQ5D
- NIH Form B
- FACT-BMT
- HAP
- PROMIS
- EORTC QLQ-C30
- Lee chronic GVHD symptom scale
- PedsQL
- CHQ
- BASES
- CHRIs
- DSI-HCT
- Other:

7.7. How do you administer routine PRO questionnaires to patients? (please check all that apply) *

- Paper-pencil
- Web-based
- Other:

7.8. How do you use routine PRO data? (outside of clinical studies, please select one per row, check all that apply) *

|  | Yes | No |
| --- | --- | --- |
| As integral part of the clinical evaluation |  |  |
| For referral to specialists  (psychiatrist, psychologist, social assistant, etc.) |  |  |
| For monitoring of response to treatment |  |  |
| For my outcome analysis |  |  |

7.9. Are you aware of any patient associations, patient support groups or patient advocacy groups active for alloHCT patients in your country? *

- Yes
- No

7.9.1. Please name the patient associations, patient support groups or patient advocacy groups offering support to HCT patients in your country *

7.9.2. Do you actively collaborate with these patient associations, patient support groups or patient advocacy groups? *

- Yes, for routine patient support only
- Yes, for research projects only
- Yes, for both routine patient support and research projects
- No

8. Does your centre perform cell-based therapies for treatment in cGVHD? *

- Yes
- No

8.1. Which cell-based therapies does your centre use in cGVHD prophylaxis if any? (please check all that apply):

- MSCs
- Tregs
- Exosomes
- ECP closed system
- ECP open system
- Not used in cGVHD prophylaxis
- Other:

8.2. Which cell-based therapies does your centre use as cGVHD treatment? (please check all that apply): *

- MSCs
- Tregs
- Exosomes
- ECP closed system
- ECP open system
- Other:

9. Are you interested in being informed about future GVHD-EUROGRAFT initiatives related to (please check all that apply): *

- GVHD epidemiology
- NIH response criteria
- PROs/QOL/Socio-economic data
- Biomarker data
- Use of cell therapy for GVHD

If yes, don't forget to provide us with your email address

10. Would you like to receive further education for your centre regarding cGVHD-related issues (please check all that apply): *

- NIH consensus diagnostic cGVHD criteria
- NIH consensus response assessment of cGVHD
- Recommendations on systemic treatments for cGVHD
- Recommendations on ancillary and supportive care for cGVHD
- Nursing issues regarding cGVHD patients
- Use of PRO/QOL tools in the post transplantation setting
- Socio-economic issues regarding cGVHD
- Other:
